# Role of ADAM33 short isoform as a tumor suppressor in the pathogenesis of thyroid cancer via oncogenic function disruption of full-length ADAM33

**DOI:** 10.1007/s13577-023-00898-3

**Published:** 2023-03-28

**Authors:** Jing Lan, Yehui Zhou, Yang Liu, Yu Xia, Yuqiu Wan, Jianbo Cao

**Affiliations:** Department of General Surgery, The first affiliated hospital of Soochow University, 188 Shizi Street, Suzhou, 215000 People’s Republic of China

**Keywords:** Thyroid cancer, ADAM33, Alternative splicing

## Abstract

Thyroid cancer is the most prevalent endocrine malignancy globally; however, its underlying pathogenesis remains unclarified. Reportedly, alternative splicing is involved in processes such as embryonic stem and precursor cell differentiation, cell lineage reprogramming, and epithelial-mesenchymal transitions. ADAM33-n, an alternative splicing isoform of ADAM33, encodes a small protein containing 138 amino acids of the N-terminal of full-length ADAM33, which constructs a chaperone-like domain that was previously reported to bind and block the proteolysis activity of ADAM33. In this study, we reported for the first time that ADAM33-n was downregulated in thyroid cancer. The results of cell counting kit-8 and colony formation assays showed that ectopic ADAM33-n in papillary thyroid cancer cell lines restricted cell proliferation and colony formation. Moreover, we demonstrated that ectopic ADAM33-n reversed the oncogenic function of full-length ADAM33 in cell growth and colony formation in the MDA-T32 and BCPAP cells. These findings indicate the tumor suppressor ability of ADAM33-n. Altogether, our study findings present a potential explanatory model of how the downregulation of the oncogenic gene *ADAM33* promotes the pathogenesis of thyroid cancer.

**Supplementary Information:**

The online version contains supplementary material available at 10.1007/s13577-023-00898-3.

## Introduction

Thyroid cancer accounts for approximately 2.5% of all diagnosed malignancies and 95% of all endocrine tumors, thereby making it the most predominant endocrine malignancy worldwide [1]. Based on different biological behaviors and pathological processes, thyroid cancer can be divided into the following four subtypes: papillary, follicular, undifferentiated, and medullary carcinoma [2]. Approximately 90% of thyroid cancers are differentiated, in which the most common histological subtype is papillary thyroid cancer (PTC). According to data from the National Cancer Institute in the United States, PTC contributes to a majority of over 56,000 new thyroid cancer cases every year [1]. Nevertheless, it has a 98% cure rate if diagnosed early and treated appropriately. Although thyroid cancer is the most curable among all malignancies, it requires research attention owing to its annually increased incidence (~ 6.3%) and mortality (~ 0.8%) [2, 3]. Studies have shown that KAP-1 [4], eIF5A2 [5], and MEIS2 [6] are involved in the progression of thyroid cancer. However, the exact pathogenesis underlying thyroid cancer remains unclear.

ADAM33 belongs to the ADAM family of membrane-anchored proteins that have a unique disintegrin and metalloprotease-containing domain structure [7]. ADAM family is highly conserved among animals from *Drosophila* to mammalian species [8]. ADAM33 was initially cloned and characterized by Yoshinaka et al. in 2002 in mouse and human tissues [7]. They found that the human ADAM33 was located on chromosome 20p13 and comprised 22 exons, which are ubiquitously expressed in tissues other than the liver. Furthermore, *ADAM33* is expressed in bronchus tissue and bronchial smooth muscle cells, rendering it a highly susceptible gene involved in asthma and other airway disorders [9–11]. In 2009, Kim et al. demonstrated that ADAM33 contributes to the pathogenesis of gastric cancer by promoting the secretion of IL-18, thereby increasing cell migration and proliferation [12]. In 2016, Stasikowska et al. revealed that ADAM33 was overexpressed in laryngeal cancer and sinonasal inverted papillomas, suggesting that ADAM33 is potentially implicated in their tumorigenesis [13]. These observations indicate that ADAM33 may be oncogenic in multiple cancer types. However, an investigation of 212 breast cancer samples indicated that *ADAM33* is silenced by DNA hypermethylation in breast cancer and that low *ADAM33* level is associated with short overall and metastasis-free survival [14]. This observation contrarily reveals that ADAM33 also possesses tumor suppressive function. Therefore, the relationship between these two entirely different functions of ADAM33 and their underlying mechanisms in cancers remain unclear.

In the present study, we aimed to investigate the function of ADAM33 in thyroid cancer. To this end, we intended to determine the effect of ectopic ADAM33 on papillary thyroid cancer cell lines and, for the first time, report the downregulated ADAM33 expression levels in thyroid cancer. The findings of our study provide information on the mechanism by which the oncogene *ADAM33* contributes to the pathogenesis of thyroid cancer.

## Materials and methods

### Patient data

From January 2016 to December 2019, 139 patients diagnosed with differentiated thyroid cancer and 91 normal controls were enrolled in the First Affiliated Hospital of Soochow University. This study included 113 papillary thyroid cancer cases and 11 follicular thyroid cancer cases. The basic clinical manifestations and baseline characteristics are summarized in Table 1. Briefly, 26–84-year-old patients (median, 54) were diagnosed and categorized into 104 stages I–II and 35 stages III–IV based on the TNM staging system [15]. Here, 83 (59.7%) patients were positive for the BRAF V600E mutant. The biopsies obtained through surgery were directly immersed into RNAlater^™^ Stabilization Solution (AM7021, Invitrogen, USA) for DNA and RNA extraction. All operations in this study were performed in accordance with the guidelines of the Declaration of Helsinki, and all experimental protocols were approved by the Ethics Committee of First Affiliated Hospital of Soochow University (no. 2022192).

**Table 1:** clinical information of THCA patients in this study

Characteristics	Total (*n* = 139)
Age, years	61(35–85)
Age at diagnosis, years	56(26–84)
Sex	
Female	66(47.5%)
Male	73(52.5%)
Histological subtypes	(0%)
Papillary	113(81.3%)
Follicular	15(10.8%)
Others	11(7.9%)
TNM Tumor stage n (%)	
*T* stage	
T1-T2	84(60.4%)
T3	45(32.4%)
T4	10(7.2%)
*N* stage	
N0	71(51.1%)
N1	68(48.9%)
*M* stage	
M0	135(97.1%)
M1	4(2.9%)
ECOG-PS	
0	72(51.8%)
1	41(29.5%)
2	26(18.7%)
Tumor size, cm	
Median (IQR)	1.0(0.7–1.5)
BRAF V600E	83 (59.7%)

TNM Tumor staging system: T, size and extent of the main tumor; N, the number of nearby lymph nodes that have cancer; M, whether cancer has metastasized (Edge et al. 2010), T0, the main tumor cannot be found; T1-4: the size and/or extent of the main tumor, N0, no cancer in nearby lymph nodes can be found; N1-3, the number and location of lymph nodes that contain cancer, M0, no spread to other parts of the body; M1, spread to other parts of the body, ECOG-PS, Eastern Cooperative Oncology Group (ECOG) performance status, IQR, interquartile range

### Cell culture and cell line construction

MDA-T32 (CRL-3351, PTC cell line) cells were purchased from the cell bank of American Type Culture Collection (ATCC). BCPAP (ACC 273, PTC cell line) cells were purchased from the cell bank of Deutsche Sammlung von Mikroorganismen und Zellkulturen. MDA-T32 and BCPAP cells were cultured in RPMI-1640 (ATCC, 30–2001) with 10% fetal bovine serum (ATCC, 30–2020), 1% glutamine (ATCC, 30–2214), and 1% mL non-essential amino acids (Gibco, 11,140–050). The cells were incubated at 37℃ in a 5% CO_2_ incubator [16].

To stably knock down ADAM33/ADAM33-short isoform expression in MDA-T32 and BCPAP cells, a modified pLKO.1 vector containing a doxycycline-induced promoter was used. The pLKO.1-sh-ADAM33/ADAM33-short isoform plasmid or pLKO.1-sh-control was transfected into the cells. Thereafter, the transfected cells were subjected to 14 days of selection in Dulbecco’s modified Eagle medium containing 1.0 μg mL^−1^ puromycin (Sigma). Lastly, the individual puromycin-resistant cells were isolated and collected for further experiments as stable expressing cells. The target sequences of ADAM33/ADAM33-short isoform shRNAs are listed in Table 2.

**Table 2:** The interference sequences and primer sequences in the study

Targets	Direct	Sequences(5'-3')
For real-time shRNA vector		
shADAM33-short#1	Forward	CCGGGGCGAGTAAGGGGCTTCCCCCTCGAGGGGGAAGCCCCTTACTCGCCTTTTTG
	Reverse	AATTCAAAAAGGCGAGTAAGGGGCTTCCCCCTCGAGGGGGAAGCCCCTTACTCGCC
shADAM33-short#2	Forward	CCGGGGGAGAGGAGGCTGGGCCTGCTCGAGCAGGCCCAGCCTCCTCTCCCTTTTTG
	Reverse	AATTCAAAAAGGGAGAGGAGGCTGGGCCTGCTCGAGCAGGCCCAGCCTCCTCTCCC
shADAM33-short#3	Forward	CCGGGGAACAAAGCGGGCATGACCCTCGAGGGTCATGCCCGCTTTGTTCCTTTTTG
	Reverse	AATTCAAAAAGGAACAAAGCGGGCATGACCCTCGAGGGTCATGCCCGCTTTGTTCC
shADAM33-full#1	Forward	CCGGGCTGCCTGCTGAAGCCGGCTCTCGAGAGCCGGCTTCAGCAGGCAGCTTTTTG
	Reverse	AATTCAAAAAGCTGCCTGCTGAAGCCGGCTCTCGAGAGCCGGCTTCAGCAGGCAGC
shADAM33-full#2	Forward	CCGGGCACCTCCTCCCACTGTCCCCTCGAGGGGACAGTGGGAGGAGGTGCTTTTTG
	Reverse	AATTCAAAAAGCACCTCCTCCCACTGTCCCCTCGAGGGGACAGTGGGAGGAGGTGC
shADAM33-full#3	Forward	CCGGGCGCATGTCCCACGCTGGAGCTCGAGCTCCAGCGTGGGACATGCGCTTTTTG
	Reverse	AATTCAAAAAGCGCATGTCCCACGCTGGAGCTCGAGCTCCAGCGTGGGACATGCGC
For real-time quantitative PCR		
GAPDH	Forward	GGAGCGAGATCCCTCCAAAAT
	Reverse	GGCTGTTGTCATACTTCTCATGG
ADAM33	Forward	CTGCTCTGGCCAGTGCCAGG
	Reverse	GCACCACTGGCTGCCCATCTG
ADAM33-short	Forward	CTGCTCTGGCCAGTGCCAGG
	Reverse	TCTGGCGGTGCATCCCAGAGC
ADAM33-full	Forward	CTTCCTGCAGTGGCGCCGGG
	Reverse	AGCCTCCACGCAGCAGCCG

### CCK8 assay for cell growth curve

The growth of MDA-T32 and BCPAP cells was determined using a cell counting kit-8 (CCK-8) assay from MedChemExpress (Cat: HY-K0301, Shanghai, China) following the manufacturer’s instructions. Briefly, the cell suspension (100 μL/well) was seeded into a 96-well plate and maintained in an incubator for 24 h. Subsequently, 10 μL CCK-8 solution was added into each well, while being careful not to generate bubbles, and the plate was then incubated for 3 h. The absorbance of each well was measured using a Synergy LX Microplate Reader at 450 nm.

### RNA isolation and real-time quantitative polymerase chain reaction (PCR) [17]

The total RNA of the cell lines and tissues was extracted using TRIzol reagent (Cat: 15,596,026, Thermo Fisher Scientific, USA) according to the manufacturer’s instructions. Next, 1 µg of total RNA was reverse-transcribed to first-strand complementary DNA (cDNA) using PrimeScript IV 1st strand cDNA Synthesis Mix (Cat: 6215A, Takara) following the manufacturer’s instructions. The cDNA products were diluted to 1/10 using ddH_2_O for real-time PCR.

Thereafter, TB Green Premix Ex Taq (Cat: RR420Q, Takara) was used in a 10 µL final volume containing 1 µL diluted cDNA. PCR involved the following steps: (1) initial denaturation at 96℃ for 5 min; (2) 40 cycles of denaturation at 96℃ for 15 s, annealing at 60℃ for 20 s, and extension at 72℃ for 20 s; and (3) melting curves in progressive heating from 65℃ to 95℃. The gene expression level of the targets was normalized to that of GAPDH. The primers involved in the real-time PCR are presented in Table 2.

### Colony formation [18]

MDA-T32 and BCPAP cell lines (10^3^ cells/well) were seeded in six-well plates. All the experiments were repeated at least thrice. Every 3 days, the medium in each well was changed until visible colonies were formed. Thereafter, the colonies were fixed using absolute methanol for 15 min and stained using 0.5% crystal violet for 30 min. Finally, the stained colonies were visualized using a Leica microscope and then quantified using the ImageJ software.

### Western blot analysis [19]

RIPA lysis buffer (Beyotime Institute of Biotechnology) was used to extract protein from the cells, and the protein concentration was determined using the BCA kit (Nanjing Jiancheng Bioengineering Inc.) according to the manufacturer’s instructions. Protein (20 μg) was then separated using 10% SDS-PAGE and transferred onto PVDF membranes (MilliporeSigma). The membranes were blocked in 5% skimmed milk for 1 h at room temperature followed by incubation with primary antibodies against the following: ADAM33 (1:2000, Cat: PA5-103,573, Thermo Fisher Scientific) and GAPDH (1:1500, Cat: HRP-60004, ProteinTech Group, Inc.) at 4℃ overnight. The membranes were then incubated with HRP-labeled goat anti-rabbit secondary antibody (Abcam, Cat: ab7090; 1:5000) at room temperature for 1 h. Thereafter, an enhanced chemiluminescence kit (Thermo Fisher Scientific) was used to determine the protein expression.

### Co-Immunoprecipitation (IP)

HA-tagged ADAM33-n plasmid (ADAM33-N-HA) was constructed by inserting full-length ADAM-33-n cDNA into pCAGGs vector (including a C-terminal HA tag). MDA-T32 and BCPAP cells were transfected with the blank and ADAM33-n-HA vector for 48 h, respectively. Co-IP was subsequently carried out using the PierceTM HA Tag IP/Co-IP kit (26,180, Thermo Fisher, MA, USA), following the producer’s protocol. Then precipitated proteins and Input were separated by SDS- PAGE and analyzed by immunoblotting with anti-ADAM33 antibody (ab113740, Abcam, Shanghai, China).

### Data mining

The differential analysis of ADAM33 expression in 512 thyroid cancer samples from the public database of the Cancer Genome Atlas (TCGA) was performed using the online bioinformatics tool gene expression profiling interactive analysis (GEPIA, http://gepia2.cancer-pku.cn/#index) [20] with a cutoff value of *p* < 0.01. Furthermore, the matched TCGA and genotype-tissue expression project (GTEx) normal tissues were used as control. The data on ADAM33 (including short and full-length isoform) expression levels in different human tissues were derived from the GTEx database (https://gtexportal.org/home/). The crystal structure of the catalytic domain of human ADAM33 (1R54) was cited from the publication of Orth et al. in 2004 [21].

### Statistical analysis

All the data are expressed as mean ± standard deviation. GraphPad software version 8.0 (San Diego, USA) was used for statistical analysis. For two sample comparisons, the Student’s *t*-test was used. One- or two-way analysis of variance was used for more than two-sample comparisons. We used the Wilson/Brown method in the receiver operating characteristic (ROC) analysis [22]. Statistical significance was set at *p* < 0.05.

## Results

### ADAM33 is downregulated in thyroid cancer

To investigate the role of ADAM33 in thyroid cancer, the GEPIA online tool was employed for the differential analysis of high throughput RNA-seq data of 512 tumors, 59 tumor-related tissues from TCGA, and 317 normal controls from the GTEx database. The results showed that ADAM33 expression in thyroid cancer was substantially decreased compared with that in the two normal controls (Figure S1A). Therefore, we collected 139 thyroid cancer samples, including 113 papillary and 15 follicular subtypes, and 11 others to validate these data using real-time PCR. Consistently, the results revealed that ADAM33 levels in tumors decreased to 49.6% (*p* < 0.001) of that in normal controls (Figure S1B). Subsequently, we performed ROC analysis to explore the possible clinical significance of ADAM33 expression levels. The results of this analysis indicated that diagnosis using ADAM33 expression to distinguish tumors from normal controls exhibited a 77.7% (95% CI: 70.1–83.8%) sensitivity and 72.5% (62.3–80.6%) specificity with an area under curve score of 0.801 (95% CI 0.750–0.862), highlighting the clinical diagnostic significance of ADAM33 (Figure S1C). Thus, it may be a potential therapeutic target for the treatment of thyroid cancer. Collectively, our findings demonstrate that ADAM33 RNA level is decreased in thyroid cancer.

### ADAM33 contributes to the pathogenesis of thyroid cancer

**Fig. 1:**
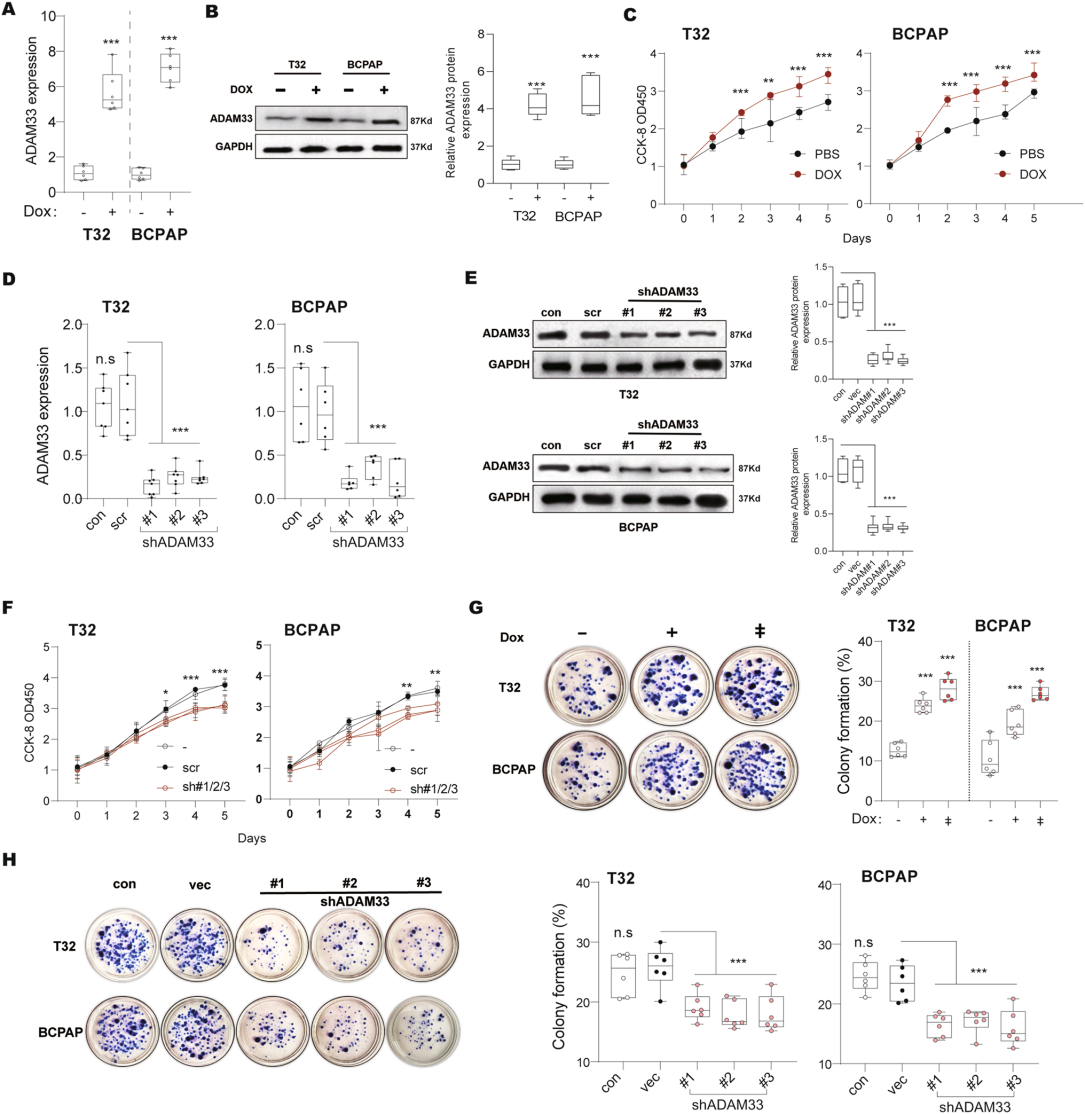
ADAM33 contributes to the pathogenesis of thyroid cancer **A** The expression level of ADAM33 in the MDA-T32 and BCPAP cells was determined by real-time PCR. Cells were treated with doxycycline at a dose of 0.1 ug/ml for 2 days. GAPDH was used as the internal control in real-time PCR. *N* = 6, ***P < 0.001 by student’s *t* test. **B** The protein level of ADAM33 in the MDA-T32 and BCPAP cells was determined by Western blot. Cells were treated with doxycycline at a dose of 0.1 ug/ml for 2 days. GAPDH was used as the internal control. *N* = 3, ***P < 0.001 by student’s t-test. **C** CCK-8 assay determined the cell viability of MDA-T32 and BCPAP cells. Cells in A were treated with doxycycline at a dose of 0.1 ug/ml since they were seeded. Every two days, the culture medium was changed. *N* = 3, **P < 0.01 and ***P < 0.001 by two-way ANOVA. **D** The expression level of ADAM33 in the MDA-T32 and BCPAP cells was determined by real-time PCR. #1/2/3 indicates an independent shRNA target. GAPDH was used as an internal control in real-time PCR. Scr, scramble shRNA; *N* = 6, n.s, no significance and ***P < 0.001 by one-way ANOVA. **E** The protein level of ADAM33 in the MDA-T32 and BCPAP cells was determined by Western blot. #1/2/3 indicates an independent shRNA target. GAPDH was used as an internal control in Western blot. Scr, scramble shRNA; *N* = 3, ***P < 0.001 by one-way ANOVA. **F** CCK-8 assay determined the cell viability of MDA-T32 and BCPAP cells. Cells in C were used. Every two days, the culture medium was changed. *N* = 3, **P < 0.01 and ***P < 0.001 by two-way ANOVA. **G-H**. Colony formation ability of ADAM33 down- and over-expressed MDA-T32 and BCPAP cells. MDA-T32 and BCPAP cell lines in A and C (10^3^ per well) were seeded in six-well plates. Every 3 days, the medium in each well was changed until the visible colonies formed. *N* = 6; n.s, no significance, ***P < 0.001 by one-way ANOVA

Considering the aberrant ADAM33 expression in thyroid cancer, we stably overexpressed its coding sequences in two PTC cell lines (MDA-T32 and BCPAP) using a doxycycline-inducible lentivector. The results of real-time PCR showed that *ADAM33* mRNA expression levels were increased to 5.3 and 7.1 fold of those in the control group after doxycycline treatment in MDA-T32 and BCPAP cells, respectively (Fig. 1A). Furthermore, ADAM33 levels showed a similar trend (Fig. 1B). Therefore, we used these two cell lines to conduct a CCK-8 assay for determining the effect of ADAM33 expression on cell growth. We observed that, since the second day, the growth of doxycycline-treated MDA-T32 and BCPAP cells was significantly faster than the control group, suggesting that ectopic ADAM33 substantially promotes cell growth (Fig. 1C). We constructed ADAM33 knockdown MDA-T32 and BCPAP cell lines to further validate these results using a modified pLKO.1 plasmid, a widely-used shRNA-delivering vector. In the MDA-T32 and BCPAP cells, *ADAM33* mRNA expression levels were largely downregulated by three independent shRNA targets, determined using real-time PCR (Fig. 1D). Additionally, ADAM33 levels were found to be decreased in the sh-ADAM33 groups (Fig. 1E). Consistently, the CCK-8 assay results indicated that ADAM33 expression decreased in response to cell growth in MDA-T32 and BCPAP cells (Fig. 1F). On performing a colony formation assay using cell lines with downregulated and over-expressed ADAM33, we observed that over-expression of ADAM33 by doxycycline treatment in MDA-T32 and BCPAP cell lines substantially increased the colony formation percentage from 12.1% to over 23.7% in a dose-dependent manner (Fig. 1G). Meanwhile, the knockdown of ADAM33 by shRNA remarkably restrained the colony formation in the MDA-T32 and BCPAP cells (Fig. 1H). Taken together, our findings demonstrate that ADAM33 possesses oncogenic function in thyroid cancer cells in vitro.

### A novel isoform of ADAM33 is aberrantly expressed in thyroid cancer samples

**Fig. 2:**
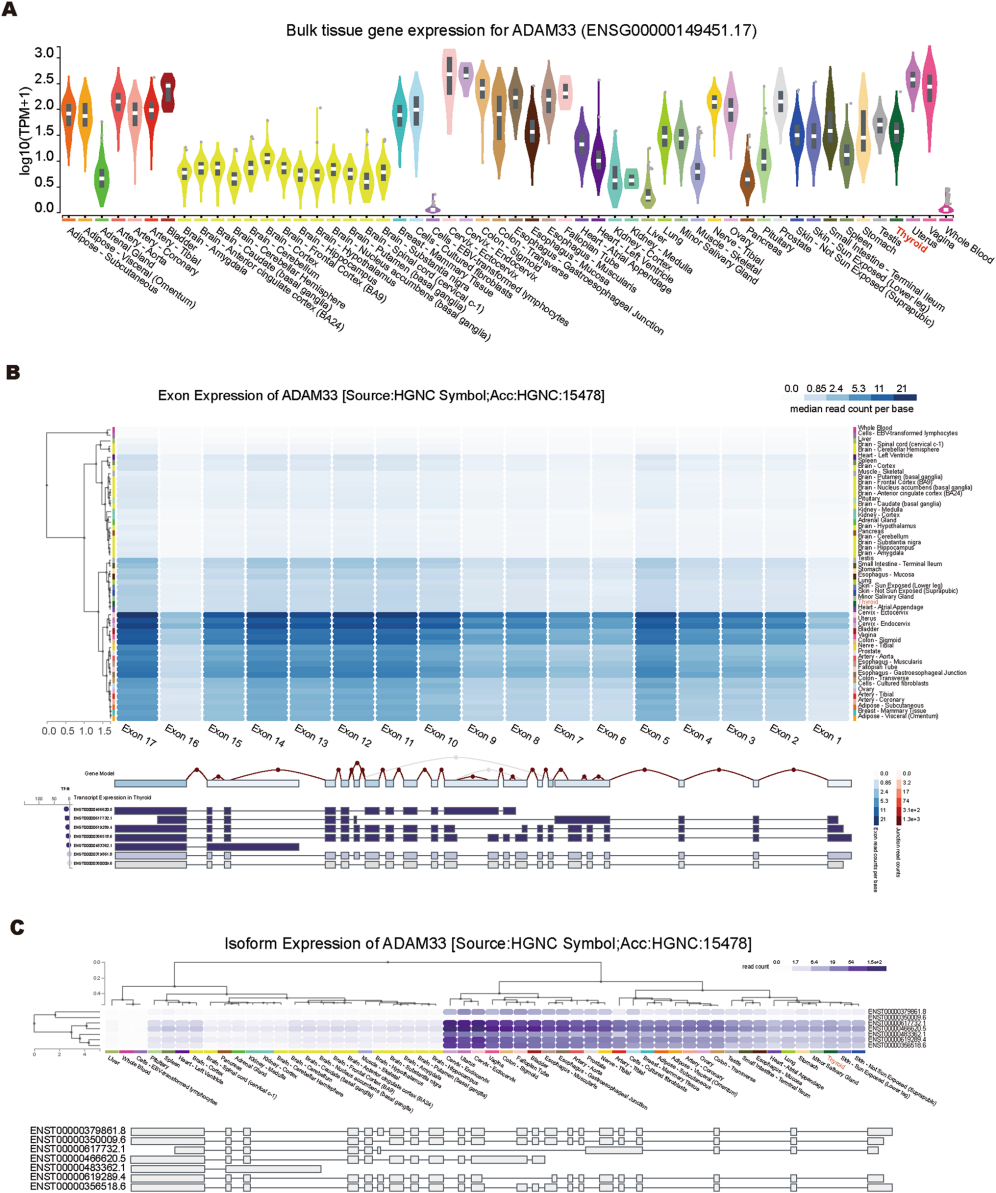
A novel isoform of ADAM33 is aberrantly expressed in thyroid cancer samples. **A** The expression level of ADAM33 in 53 different human tissues. The expression data are derived from the GTEx database. TPM, Transcripts Per Million mapped reads. **B** The usage of ADAM33 exons in 53 different human tissues. The expression data are derived from the GTEx database.** C** The expression pattern of ADAM33 isoforms in 53 different human tissues. The expression data are derived from the GTEx database

A contradiction exists between ADAM33 downregulation in thyroid cancer biopsy samples and its oncogenic function in thyroid cancer cells, and its underlying mechanism remains unelucidated. To this end, we systemically analyzed ADAM33 expression in 53 types of human tissue samples using RNA-seq data from the GTEx database. ADAM33 was ubiquitously and highly expressed in almost all human tissues except the brain (Fig. 2A). Powell et al. performed an analysis of alternatively spliced isoforms of ADAM33 in primary human airway fibroblasts in 2004; they demonstrated that the different forms are located both in the nucleus and cytoplasm [23]. In 2005, Haitchi et al. reported several *ADAM33* mRNA splice variants in bronchial biopsies and embryonic lungs using PCR, which was further confirmed using western blotting [24]. These findings indicate that different ADAM33 isoforms may exhibit diverse functions in thyroid cancer. Therefore, we analyzed the expression pattern of ADAM33 in 53 different human tissues using the RNA-seq data from the GTEx database. As shown in Fig. 2B, the usage frequency of different exons in ADAM33 is inhomogeneous among tissue types, suggesting that alternative splicing isoforms of ADAM33 are common. Notably, the top two highly expressed isoforms were ENST00000466620 and ENST00000617732, and not the full-length ENST00000356518 (Fig. 2B and C). Concerning protein-coding potential, the transcript NST00000466620 does not have a coding protein, whereas ENST00000617732 codes for a protein with 138 amino acids. Therefore, we focused on the role of the transcript ENST00000617732 in thyroid cancer; this transcript was named ADAM33-n based on its amino acid sequences. We quantified the ADAM33-n expression level in the collected thyroid cancer biopsies and found that the aberration of *ADAM33* in tumors is primarily attributable to the downregulation of ADAM33-n (Figure S1D). Moreover, we compared the expression level of ADAM33-n and full-length ADAM33 (Figure S2), and the results showed that the ADAM33-n level was more dominant than the full-length one.

### ADAM33 short isoform exhibits anti-oncogenic roles in thyroid cancer

**Fig. 3:**
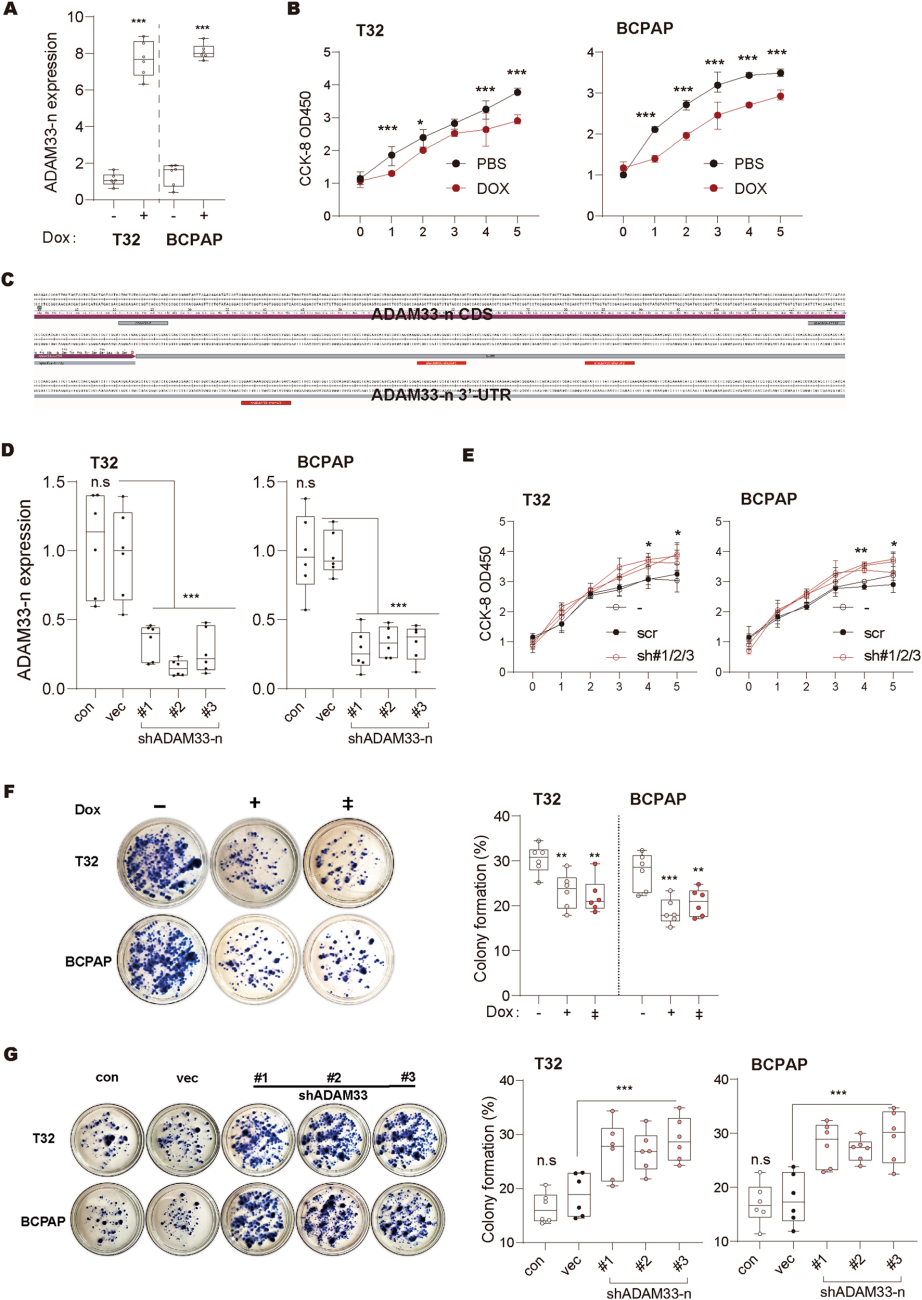
ADAM33 short isoform exhibits anti-oncogenic roles in thyroid cancer. **A** The expression level of ADAM33-n in the MDA-T32 and BCPAP cells was determined by real-time PCR. Cells were treated with doxycycline at a dose of 0.1 ug/ml for 2 days. GAPDH was used as an internal control in real-time PCR. *N* = 6, ***P < 0.001 by student’s *t* test. **B** CCK-8 assay determined the cell viability of MDA-T32 and BCPAP cells. Cells in A were treated with doxycycline at a dose of 0.1 ug/ml since they were seeded. Every two days, the culture medium was changed. *N* = 3, *P < 0.05 and ***P < 0.001 by two-way ANOVA. **C** The location of ADAM33-n specific shRNAs. CDS, coding sequence, 3’-UTR, 3’-untranslated region.** D** The expression level of ADAM33 in the MDA-T32 and BCPAP cells we collected was determined by real-time PCR. #1/2/3 indicates an independent shRNA target. GAPDH was used as an internal control in real-time PCR. Scr, scramble shRNA; *N* = 6, n.s, no significance and ***P < 0.001 by one-way ANOVA. **E** CCK-8 assay determined the cell viability of MDA-T32 and BCPAP cells. Cells in D were used. Every two days, the culture medium was changed. *N* = 3, *P < 0.05 and **P < 0.01 by two-way ANOVA. **F-G**. Colony formation ability of ADAM33 down- and over-expressed MDA-T32 and BCPAP cells. MDA-T32 and BCPAP cell lines in A and D (10^3^ per well) were seeded in six-well plates. Every 3 days, the medium in each well was changed until the visible colonies formed. *N* = 6; n.s, no significance, **P < 0.01 and ***P < 0.001 by one-way ANOVA

To explore the role of ADAM33-n in the pathogenesis of thyroid cancer, we stably transfected the coding sequences of ADAM33-n in MDA-T32 and BCPAP cells using a doxycycline-inducible lentivector. After doxycycline treatment, real-time PCR results revealed that ADAM33-n expression level was upregulated to 7.9 and 8.1 fold of that in the control in MDA-T32 and BCPAP cells, respectively (Fig. 3A). By contrast, we observed that doxycycline treatment on these two cell lines significantly inhibited cell growth compared with that in the PBS group, determined using the CCK-8 assay (Fig. 3B). We further validated the findings using ADAM33-n knockdown cell lines. Therefore, we designed three independent shRNA for ADAM33-n transcripts at its 3′-UTR, which is distinct from other transcripts in sequences (Fig. 3C). These three shRNAs were stably transfected into MDA-T32 and BCPAP cells using pLKO.1 plasmid. Real-time PCR results indicated that ADAM33-n was specifically knocked down without interfering with full-length ADAM expression (Fig. 3D). Similarly, with ADAM33-n overexpressed cells, the CCK-8 assay revealed that downregulation of the short isoform of ADAM33 enhanced cell growth ability compared with that in the scramble group (Fig. 3E). Furthermore, we performed a colony formation assay using the ADAM33-n downregulated and over-expressed cell lines mentioned earlier. Our observations showed that ectopic ADAM33-n induced by doxycycline treatment substantially decreased the colony formation percentage from 30.8% to less than 24.5% in MDA-T32 and BCPAP cell lines in a dose-dependent manner (Fig. 3F). Meanwhile, down-regulation of ADAM33-n using shRNA substantially promoted colony formation both in MDA-T32 and BCPAP cells (Fig. 3G). Collectively, our data demonstrated that, unlike full-length ADAM33, ADAM33-n is a tumor suppressor in thyroid cancer cells in vitro.

### ADAM33 short isoform interferes with the oncogenic function of full-length ADAM33

**Fig. 4:**
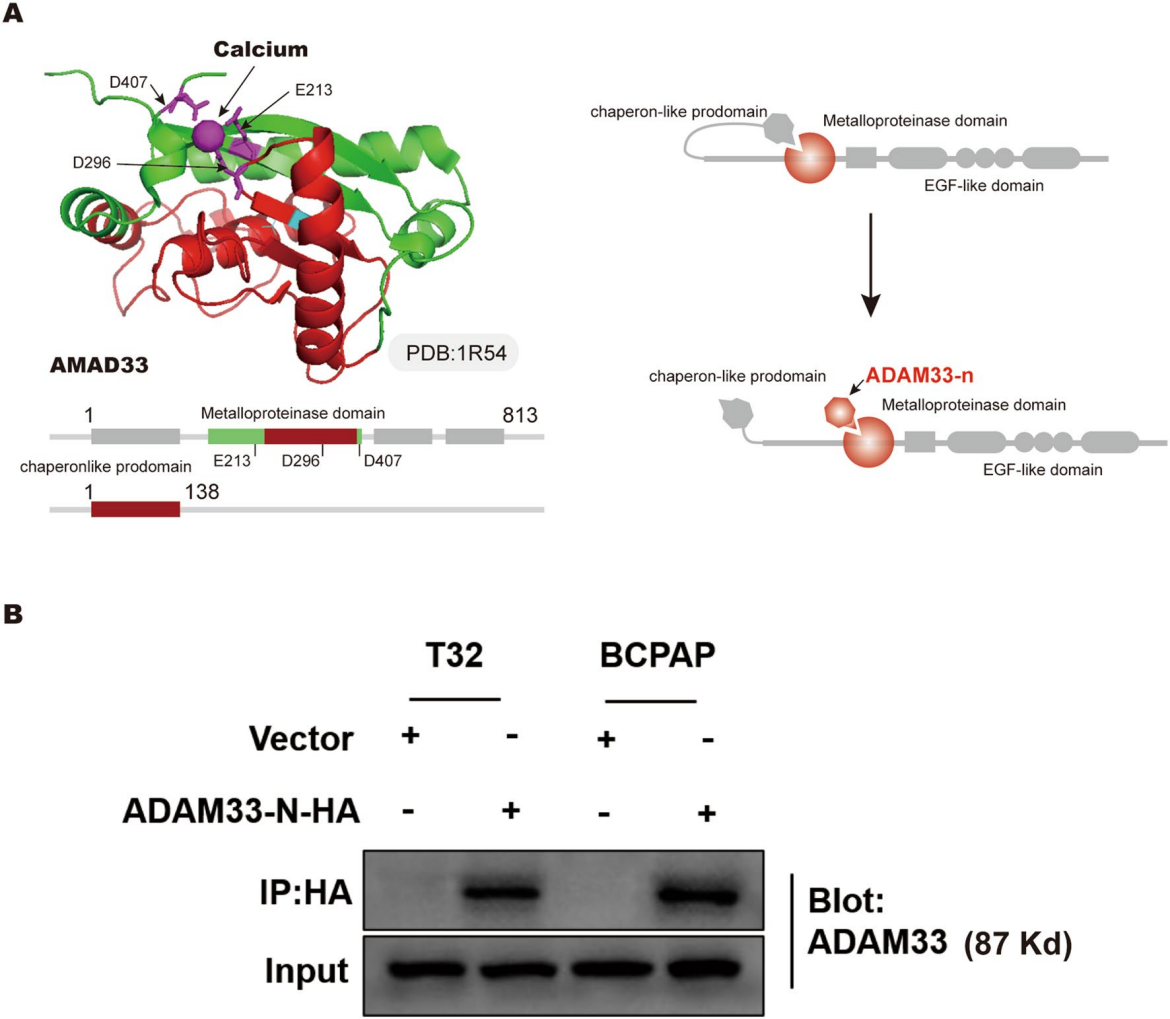
ADAM33 interacts with ADAM33-n. **A** Crystal structure of ADAM33 catalytic domain (left) and the putative mechanism of ADAM33-n interfering full-length ADAM33 function. The carton of ADAM33 catalytic was generated based on the published structure in the PDB database (1R54).** B** Co-immunoprecipitation of HA-tagged ADAM33-n with ADAM33 in MDA-T32 and BCPAP cells

**Fig. 5:**
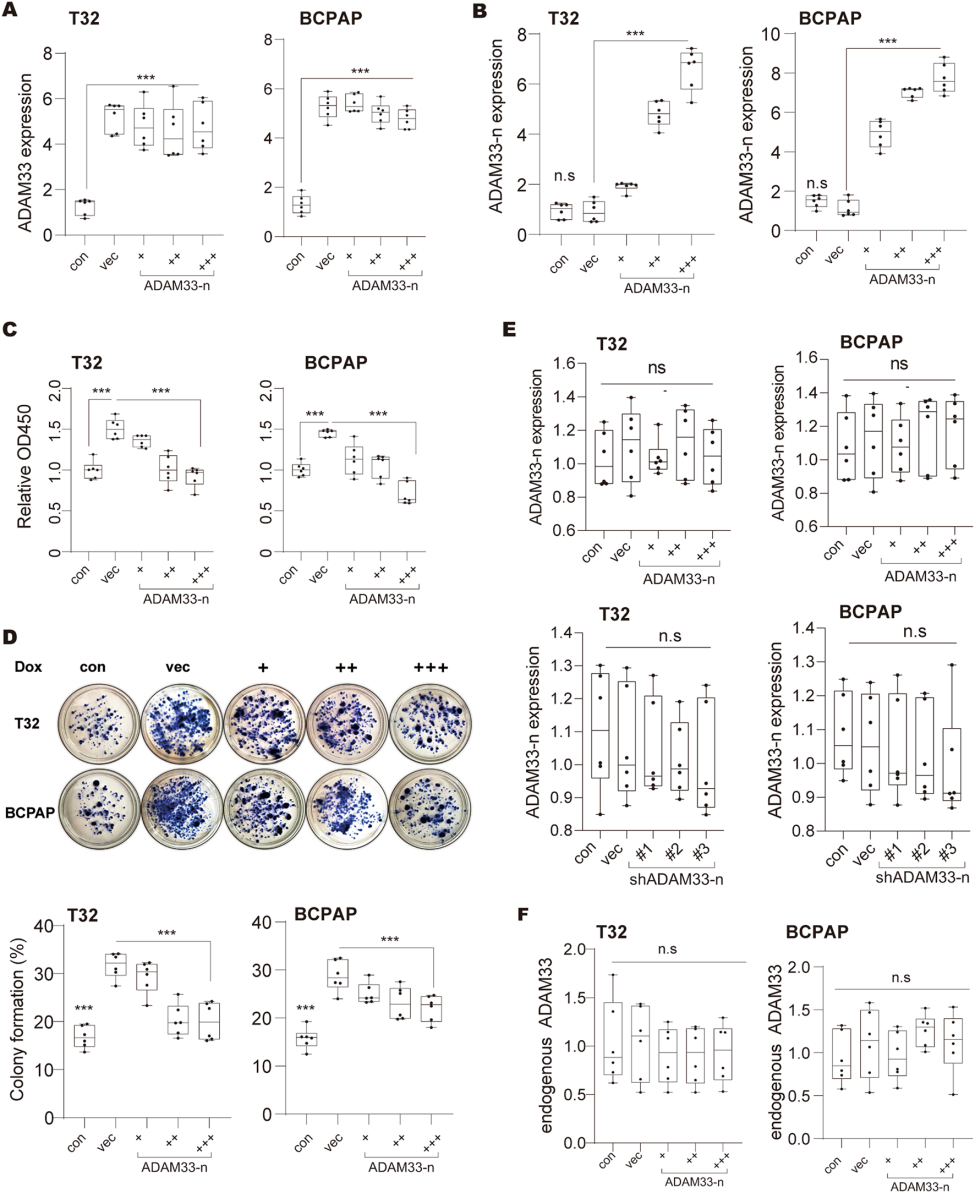
ADAM33 short isoform interfere the oncogenic function of full length ADAM33. **A-B** The expression level of ectopic ADAM33 **B**/ADAM33-n **C** in the MDA-T32 and BCPAP cells we collected was determined by real-time PCR. GAPDH was used as an internal control in real-time PCR. VEC, empty vector; *N* = 6, ***P < 0.001 by one-way ANOVA. **C** CCK-8 assay determined the cell viability of MDA-T32 and BCPAP cells. Cells in B and C were used. Every two days, the culture medium was changed. *N* = 3,***P < 0.001 by two-way ANOVA. **D** Colony formation ability of ADAM33 down- and over-expressed MDA-T32 and BCPAP cells. MDA-T32 and BCPAP cell lines in B and C (10^3^ per well) were seeded in six-well plates. Every 3 days, the medium in each well was changed until the visible colonies formed. *N* = 6; ***P < 0.001 by one-way ANOVA. **E**. The expression of ADAM33-n when ADAM33 was overexpressed or knocked down. VEC, empty vector; *N* = 6, n.s, no significance by one-way ANOVA. **F**. The expression level of endogenous ADAM33 in the MDA-T32 and BCPAP cells we collected was determined by real-time PCR. Cells were stably over-expressed with ADAM33-n in a concentration gradient. GAPDH was used as an internal control in real-time PCR. VEC, empty vector; *N* = 6, n.s, no significance by one-way ANOVA

ADMA33 is a type I transmembrane zymogen glycoprotein, which belongs to the family of disintegrin and metalloprotease [25]. ADAM33 protein comprises several domains, such as pro-metalloprotease, cysteine-rich, disintegrin-like, transmembrane, EGF-like, and cytoplasmic domains, which facilitate many critical biological processes, including cell activation, adhesion, proteolysis, signaling, and fusion [26–29]. In the quiescent status, a chaperone-like prodomain in the amino-terminal extracellular fragment of ADAM33 binds to the metalloproteinase domain to inhibit the proteolytic activity of ADAM33 [21, 30] (Fig. 4A). Therefore, we speculated that the ADAM33-n isoform may form a chaperon-like protein that directly binds to the metalloproteinase domain of full-length ADAM33, and unlike that of the chaperone-like prodomain, the inhibitory effect of ADAM33-n could not be reversed (Fig. 4A). In addition, in order to explore the direct interaction between full-length ADAM33 and ADAM33-n, we expressed HA-tagged ADAM33-n in MDA-T32 and BCPAP cells, and performed a Co-IP assay. The results clearly showed the interaction between full-length ADAM33 with ADAM33-n (Fig. 4B). Thus, the ADAM33-n isoform may be a constitutive inhibitor of full-length ADAM33. To validate this hypothesis, we co-transfected ADAM33-n and full-length ADAM33 in MDA-T32 and BCPAP cells, and ADAM33-n was over-expressed in a concentration gradient. The results of real-time PCR revealed that full-length ADAM33 and ADAM33-n levels increased to about 5.1 and 1.9–7.3 folds, respectively, of those in the control group (Fig. 5A and B). In the CCK-8 assay, we observed that the elevated cell growth ability by ADAM33 over-expression in MDA-T32 and BCPAP cells was substantially reversed by ectopic ADAM33-n in a dose-dependent manner (Fig. 5C). Furthermore, the colony formation assay results revealed that ectopic ADAM33-n overcame the oncogenic effect of full-length ADAM33 in MDA-T32 and BCPAP cells in vitro (Fig. 5D). In addition, when we overexpressed or knocked down only ADAM33 in MDA-T32 and BCPAP cells, the expression of ADAM33-n mRNA was not significantly changed (Fig. 5E). In contrast, when we transfected only ADAM33-n in MDA-T32 and BCPAP cells, the over-expression of ADAM33-n failed to influence the endogenous full-length *ADAM33* mRNA level (Fig. 5F). Our results demonstrated that the ADAM33 short isoform interferes with the oncogenic function of full-length ADAM33 without influencing its mRNA level.

## Discussion

During the maturation of pre-RNA precursors, alternative splicing is a critical posttranscriptional step to ensure that one gene produces multiple mature mRNAs that are ultimately translated into different proteins [31, 32]. The pervasive cellular process of alternative splicing expands the utilization efficiency of the genome to contribute to proteome complexity [33, 34]. Among higher eukaryotes, alternative splicing is frequently implicated in modulating the patterns of gene expression that play a critical role in cell fate decisions [35]. However, aberration or errors in alternative splicing often produce a deleterious impact on cells and even lead to cell death as well as cancerization [36, 37]. Alternative splicing events are regarded to be key markers of tumor progression and prognosis, including those of bladder [38] and liver cancer [39]. Lin et al. performed survival analysis in 496 patients with PTC and found that 2799 splicing events harbor prognostic significance in distinguishing TNM stage, tumor stage, distant metastasis, and tumor status of papillary thyroid cancer [40].

In 2009, Kim et al. reported that ADAM33 is implicated in the pathogenesis of gastric cancer and that its overexpression results in increased cell migration and proliferation [12]. In 2017, Manica et al. showed that ADAM33 is downregulated in breast tumor samples (*n* = 212) and that its low levels are associated with triple-negative breast cancer, basal-like markers, and shorter overall survival [14]. In our study, the real-time PCR results of 139 thyroid cancer biopsy samples support that ADAM33 is downregulated in tumor tissues. This observation gives rise to the following question: how does the downregulation of an oncogene promote cell growth or proliferation? To investigate this, we systematically analyzed the alternative transcripts of ADAM33 using the RNA-seq data of 53 different human tissues from the GTEx database. We found that the alternative splicing isoform of ADAM33, ENST00000617732 (ADAM33-n), was ubiquitously and highly expressed in most human tissues, comparable to that of the full-length ADAM33. Therefore, we verified the real-time PCR primers for ADAM33 and found that the ones we used can target the exons 15 and 16 of ADAM33, indicating that they can detect the expression of almost all transcripts. Accordingly, we suspected that ADAM33-n expression may be dysregulated in thyroid cancer. After determining its expression in the collected thyroid cancer biopsies, we observed that ADAM33-n was downregulated in tumors compared with the normal controls. Additionally, we compared the expression level of ADAM33-n and full-length ADAM33 (Figure S2), and the results showed that the ADAM33-n level is more dominant than the full-length one. These findings demonstrate that ADAM33 is the dominant contributor to the aberration of *ADAM33* in thyroid cancer.

Reportedly, alternative splicing networks occur in numerous processes such as embryonic stem and precursor cell differentiation, cell lineage reprogramming, and epithelial-mesenchymal transitions [41–43]. For instance, *MAP4K4* mRNA was reported to be alternatively spliced in papillary thyroid cancer samples by RBM17, which causes phosphorylation of downstream signaling pathways [44, 45]. Structurally, ADAM33-n loses exons 5–12 of full-length ADAM33 and encodes only a small N-terminal peptide (1–138 amino acids) that reserves the chaperone-like prodomain. As the chaperon-like prodomain of ADAM33 binds to the catalytic domain to inhibit its proteolytic activity, we hypothesized that ADAM33-n is a natural inhibitor of full-length ADAM33 and that the downregulation of ADAM33-n consequently restores the oncogenic function of ADAM33 in thyroid cancer (Fig. 4A). We observed that ectopic ADAM33-n overcame the effect of ADAM33 overexpression in cell growth and colony formation in MDA-T32 and BCPAP cells in a dose-dependent manner. Moreover, our results revealed that ectopic ADAM33-n in MDA-T32 and BCPAP cells failed to influence the expression level of endogenous ADAM33, implying that ADAM33-n may interfere with the oncogenic full-length ADAM33 at the protein level. This observation is consistent with our hypothesis. Therefore, we concluded that ADAM33-n acts as a tumor suppressor by blocking the oncogenic role of full-length ADAM33 in thyroid cancer.

In summary, we found that ADAM33-n, an N-terminal isoform of ADAM33, is the dominant contributor to *ADAM33* aberration in thyroid cancer. Unlike the full-length ADAM33, ectopic ADAM33-n inhibits cell growth and colony formation, indicating its tumor suppressor ability. Altogether, our study findings demonstrate how the downregulation of an oncogenic gene, *ADAM33*, promotes the pathogenesis of thyroid cancer, thereby indicating its potential as a therapeutic target. However, our study has some limitations. Further experiments are warranted to validate the function and relationship of ADAM33 and ADAM33-n and to substantiate the results based on tumor samples and clinical data.

## Supplementary Information

Below is the link to the electronic supplementary material.

Supplementary file1 Fig. S1 ADAM33 is down-regulated in thyroid cancer. A RNA-seq data in the public database showed the down-regulation of ADAM33 in tumors. Gene expression profiling interactive analysis (GEPIA) online tool was employed for the differential gene analysis. THCA, thyroid cancer from TCGA, GTEx, the database of Genotype-Tissue Expression Project. P-value < 0.01 sets as the cutoff, by one-way ANOVA. B The expression level of ADAM33 in the clinical biopsies we collected was determined by real-time PCR. 91 normal controls and 139 patients with thyroid cancer were involved. GAPDH was used as an internal control in real-time PCR. ***P < 0.001 by student’s t-test. CThe ROC curve in distinguishing thyroid cancer from normal participants based on ADAM33 expression in RNA level. ROC, receiver operating curve; AUC, area under curve in ROC analysis; CI, confidential interval. Wilson/Brown method was used for statistical analysis. The expression level of ADAM33-n isoform in the clinical biopsies we collected was determined by real-time PCR. Samples are the same as in A. ***P < 0.001 by student’s t-test. (TIF 1255 KB)

Supplementary file2 Fig. S2 The expression level of ADAM33-n and full-length ADAM33. The compared results were converted to -log2 (discrete cosine transform). ***P < 0.001 by one-way ANOVA. (TIF 40 KB)

## Data Availability

The data in this research are available upon request from the corresponding author.
