# Supplementary figures and images for: Role of ADAM33 short isoform as a tumor suppressor in the pathogenesis of thyroid cancer via oncogenic function disruption of full-length ADAM33
Source: Hum Cell. 2023 Mar 28;36(4):1451–63. doi: 10.1007/s13577-023-00898-3 (PMC10284970; doi:10.1007/s13577-023-00898-3)

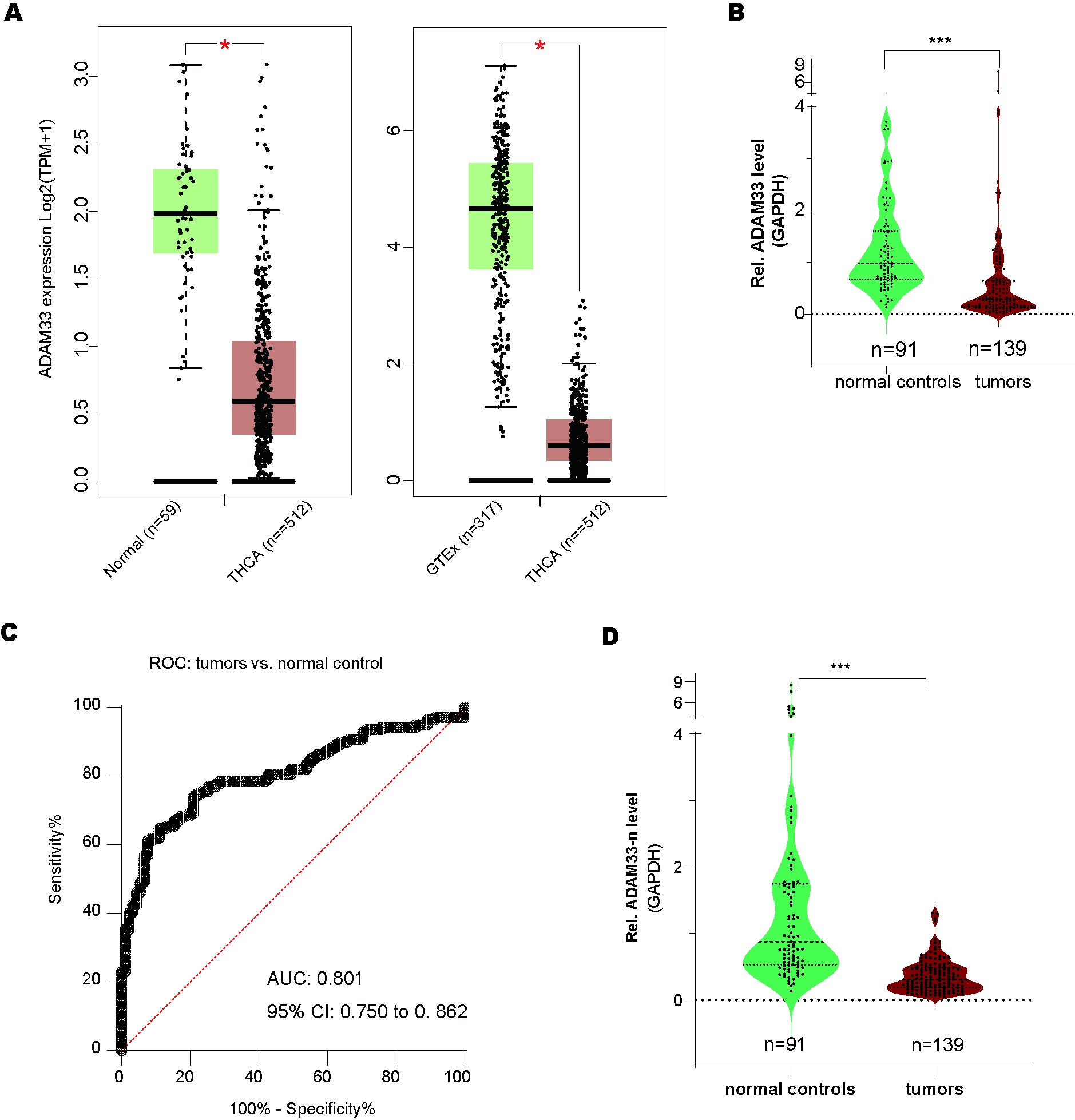

Supplement: Supplementary file 1 — Supplementary file1 Fig. S1 ADAM33 is down-regulated in thyroid cancer. A RNA-seq data in the public database showed the down-regulation of ADAM33 in tumors. Gene expression profiling interactive analysis (GEPIA) online tool was employed for the differential gene analysis. THCA, thyroid cancer from TCGA, GTEx, the database of Genotype-Tissue Expression Project. P-value < 0.01 sets as the cutoff, by one-way ANOVA. B The expression level of ADAM33 in the clinical biopsies we collected was determined by real-time PCR. 91 normal controls and 139 patients with thyroid cancer were involved. GAPDH was used as an internal control in real-time PCR. ***P < 0.001 by student’s t-test. CThe ROC curve in distinguishing thyroid cancer from normal participants based on ADAM33 expression in RNA level. ROC, receiver operating curve; AUC, area under curve in ROC analysis; CI, confidential interval. Wilson/Brown method was used for statistical analysis. The expression level of ADAM33-n isoform in the clinical biopsies we collected was determined by real-time PCR. Samples are the same as in A. ***P < 0.001 by student’s t-test. (TIF 1255 KB) [file 13577_2023_898_MOESM1_ESM.tif]

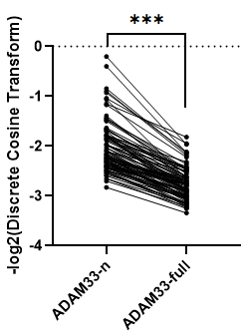

Supplement: Supplementary file 2 — Supplementary file2 Fig. S2 The expression level of ADAM33-n and full-length ADAM33. The compared results were converted to -log2 (discrete cosine transform). ***P < 0.001 by one-way ANOVA. (TIF 40 KB) [file 13577_2023_898_MOESM2_ESM.tif]
